# The genetic basis of mood and anxiety disorders – changing paradigms

**DOI:** 10.1186/2045-5380-2-17

**Published:** 2012-10-01

**Authors:** Elisabeth B Binder

**Affiliations:** 1 Max-Planck Institute of Psychiatry, Munich Germany and Dept. of Psychiatry and Behavioral Sciences Emory University School of Medicine, Atlanta, GA, USA

## Abstract

Family, twin and epidemiologic studies all point to an important genetic contribution to the risk to develop mood and anxiety disorders. While some progress has been made in identifying relevant pathomechanisms for these disorders, candidate based strategies have often yielded controversial findings. Hopes were thus high when genome-wide genetic association studies became available and affordable and allowed a hypothesis-free approach to study genetic risk factors for these disorders. In an unprecendented scientific collaborative effort, large international consortia formed to allow the analysis of these genome-wide association datasets across thousands of cases and controls ([1] and see also http://www.broadinstitute.org/mpg/ricopili/). Now that large meta-analyses of genome-wide association studies (GWAS) have been published for bipolar disorder and major depression it has become clear that main effects of common variants are difficult to identify in these disorders, suggesting that additional approaches maybe needed to understand the genetic basis of these disorders [2,3].

## 

Several factors need to be considered when evaluating the results of genetic association studies in mood and anxiety disorders.

## Phenotype definition

In contrast to other medical disorders, diagnoses for psychiatric disorders are still mostly based on verbal report and observation and not biological measures. It is thus very likely that our symptom-based diagnoses include patients with different primary pathophysiological disturbances and thus likely predisposing genetic variants reducing the power of genetic association studies. In addition, our diagnostic scheme may separate individuals with common genetic risk factors into different categories. For example, it is likely that bipolar disorder patients share genetic susceptibility loci with patients with major depression as well as schizophrenia [4]. Specific symptom categories are shared across mood and anxiety disorders and the use of observational dimensional behavioural phenotypes that cut across diagnostic categories such as psychotic or anxiety symptoms may allow to identify new risk genes for psychiatric disorders [5]. In addition, intermediate phenotype or endophenotypes in mood and anxiety disorders may be a promising approach, as it is likely that the effect sizes for genetic associations with biological measures are greater than with more remote psychiatric diagnoses. Such phenotypes include but are not limited to neuroimaging, endocrine and neurophysiological measures [6]. In the future, these phenotypes could even include *in vitro* observations on neurons derived from inducible pluripotent stem cells from patient fibroblasts [7].

## Functional annotation of associated variants

The published GWAS in mood disorders all highlight that a high number of common variants with small odd ratios likely cumulatively contribute to the risk to suffer from these disorders [8]. While this implies that very large samples will be needed to robustly detect these variants, it also indicates that true associations may not reach genome-wide significance [9]. This has spurred a number of re-analyses of GWAS for psychiatric disorders that explore whether functionally relevant genetic variants maybe be over-represented among the nominally significant associations and whether this overlap could highlight specific pathways relevant for the disease. To date, investigators have mostly focused on using expression quantitative trait locus (eQTL) analysis as well as methylation quantitative trait locus (mQTL) analysis in different tissues to narrow in on putatively functionally relevant polymorphisms, i.e. variants associated with different gene expression or DNA methylation levels [10].

In addition, combining genome-wide protein and gene expression datasets and studies in animal models of mood and anxiety disorders with human genetic studies may also help to identify relevant candidate genes from GWAS. For example, combining GWAS and gene expression studies for bipolar disorder, association signals appear to cluster in discrete regions of the protein–protein interaction network, showing replicated evidence for association for networks involving several interlinked signaling pathways, such as transmission of nerve impulse, Wnt and Notch signaling [11]. Such analyses highlight the importance of investigating such datasets not only on the single gene level but also on the level of functional pathways and new tools allow for stringent testing of the enrichment of signals in specific pathways [12]. Such pathway based analyses also allow better to integrate data from a number of different sources, such as gene expression and proteome datasets, GWAS, copy number variation or rare variant analyses that can highlight overlapping signals from different types of studies [13].

## Common vs. rare variants

For schizophrenia and autism but also bipolar disorder it has become increasingly clear that rare variants in addition to common variants contribute to the risk of disease [14]. New technological developments, including next generation sequencing will allow us to study large numbers of patients for the presence of rare functional variants and data from re-sequencing effort of the complete RNA coding sequence (exome) are starting to be published [15]. However, these studies indicate that very large samples, with over 10,000 individuals will be required for sufficient power [16].

## Gene x environment interactions

Finally, the environmental risk component is strong in mood and anxiety disorders, especially major depression. A number of candidate gene studies suggest that genes might moderate the effects of environmental risk factors in the absence of main genetic effects [17]. GWAS may profit from the inclusion of environmental exposure as a variable and this could unmask a number of important genetic moderator factors that are not apparent when combining cases and control with different exposure levels to risk environments. However, genome-wide gene x environment studies will require larger sample sizes and will have to deal with the problem of reliably quantifying environmental exposure in studies recruiting from different populations, especially when using retrospective measures. Here, the use of epigenetic investigations in peripheral blood cells may prove useful as biomarker for environmental exposure [18].

## Conclusion

In conclusion, while the results from genetic studies in mood and anxiety disorders may seem disappointing at first sight, a panoply of new analytic strategies and molecular genetic tools is likely to yield significant discoveries in the near future that further our understanding of the pathophysiology of these disorders, some of which will be highlighted in this series.
